# Nanoparticle-Infused UHMWPE Layer as Multifunctional Coating for High-Performance PPTA Single Fibers

**DOI:** 10.1038/s41598-019-43629-1

**Published:** 2019-05-09

**Authors:** Zhuolei Zhang, Yao Zhao, Haoqi Li, Simona Percec, Jie Yin, Fei Ren

**Affiliations:** 1 Department of Mechanical Engineering, Temple University, Philadelphia, PA 19122 USA; 2 Temple Materials Institute, Temple University, Philadelphia, PA 19122 USA

**Keywords:** Polymers, Polymers, Mechanical engineering, Mechanical engineering, Mechanical engineering

## Abstract

High-performance fibers made of poly-(*p*-phenylene terephthalamide) (PPTA) with high stiffness and high strength are widely used in body armor for protection due to their high degree of molecular chain alignment along the fiber direction. However, their poor mechanical properties in the transverse direction and low surface friction are undesirable for applications requiring resistance to ballistic impact. Here we provide a simple yet effective surface engineering strategy to improve both the transverse mechanical properties and the tribological property by coating PPTA fibers with ultra-high molecular weight polyethylene (UHMWPE) embedded with silica nanoparticles. The coated-PPTA fiber shows remarkable enhancement in transverse mechanical properties including ~127% increase of Young’s modulus, which is attributed to both the alignment of UHMWPE chains in the transverse direction and the embeded ceramic nanoparticles. Meanwhile, the surface friction of the coated fiber increases twofold as a result of the ceramic nanoparticles. In addition, the coated fibers exhibit an enhanced chemical resistance to external harsh environment. The improved transverse mechanical properties, surface frictional characteristics, and chemical resistance demonstrate that coating with UHMWPE and ceramic nanoparticles can be used as an effective approach to enhance the performance of PPTA and other high-performance polymer fibers for body armor applications.

## Introduction

Poly(*p*-phenylene terephthalamide) fibers (PPTA fibers) have been extensively used to fabricate impact-resistant fabrics owing to their extraordinary mechanical properties including high specific tensile strength, high toughness, and excellent energy absorbing ability^1–6^. To fabricate armor materials against ballistic impact, PPTA fibers are often woven into fabrics, consisting of interlocked fibers^7–9^. When a projectile strikes a fabric, the fibers experience not only severe tensile deformation along the longitudinal direction but also substantial transverse compressive stress. Dynamically, the projectile is caught in a web of fibers to which its kinetic energy is transferred. This kinetic energy, carried by the stress waves at the striking point, is dissipated through fiber deformations and inter-fiber friction when slipping or sliding against each other. Thus, both mechanical properties and frictional properties of single fibers play an important role in the overall impact resistant performance of body armor materials^10–12^. However, to date, most studies focus on the mechanical properties of single fibers along their longitudinal direction^13–18^. Compared to the covalent bonds and extra inter-chain bonds along the longitudinal direction^4,13–15^, the hydrogen bonds and aromatic stacking interactions in the transverse direction are much weaker. Consequently, the poor mechanical properties along the transverse direction would potentially deteriorate their protective performance due to material damage and failure^16–19^. Thus, strengthening the transverse compression properties of PPTA fibers is highly needed, which remains largely unexplored.

To date, a variety of methods or strategies are proposed to enhance either the transverse mechanical properties or the frictional characteristics. A common method to improve the fiber’s transverse mechanical properties is thermal treatment. For example, Sweeney *et al*.^20,21^ reported that thermal treatment of Kevlar-29 fiber could lead to a significant enhancement (nearly 50%) of the transverse strength of the fiber. However, this would be accompanied by approximately 20% reduction in tensile strength, due to a combination of misorientation of crystallites induced by hydrogen bond disruptions within the core region and free-radical formation induced crosslinking in the skin region. In order to enhance the surface frictional characteristics, several efforts have been made, for example, by coating the fabrics with shear thickening fluids, modifying the surface via plasma treatment, and coating the fibers with rubbery materials possessing higher friction coefficient^22–28^. However, it is still a significant challenge to simultanously increase the transverse mechanical property and enhance the surface frictional characteristics of the PPTA fibers, which is highly desired for advanced ballistic applications.

In additon to mechanical properties, the chemical robustness of fibers is also an important scenario to be considered since the body armors may be exposed to harsh environmental conditions. Under some specific external chemical attacks (e.g., acidic or alkaline conditions), significant deterioration of the fibers due to bond breaking could result in a weakening of their mechanical strength^4,13^. Therefore, improvement of mechanical properties in the transverse direction and chemical stability of PPTA fibers are highly desired for their protective applications.

To achieve the above-mentioned objectives, here we propose a new polymer dip-coating strategy to coat the PPTA fibers with highly crystalliized ultra-high molecular weight polyethylene (UHMWPE) and ceramic nanoparticles to improve both the transverse properties and the surface friction. UHMWPE is chosen due to its good mechanical properties, good processability (thermoplastic), remarkable strength-to-weight ratio, and an excellent energy absorption ability^29,30^. The Young’s modulus of UHMWPE could exceed 200 GPa when its crystallinity is close to 99%^31^. In addition, the relatively low melting point of UHMWPE (~135 °C) enables processing at relatively low temperatures.

## Results and Discussion

In this paper, we found that coating of UHMWPE on PPTA fibers results in a remarkable enhancement in trasverse mechanical properties. We also explored the incorporation of SiO_2_ nanoparticles into the UHMWPE, which led to further significant enhancement of mechanical properties including Young’s modulus and hardness along the transverse direction and about 100% higher surface friction coefficient. In addition, the coated PPTA fibers exhibit strong resistance to external harsh environments. Results from this paper provides a new engineering route to enhance the mechanical performance of high-strength PPTA based fibers.

**Figure 1:**
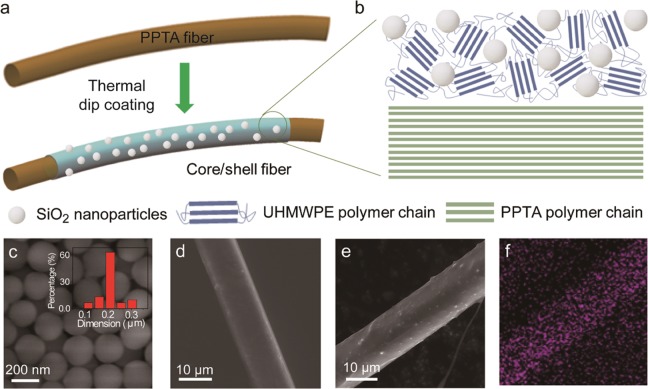
(**a**) Schematic illustration of coating a PPTA fiber with UHMWPE and SiO_2_ nanoparticles. (**b**) Hypothetical structures of the PPTA fiber and the coating. (**c**) An SEM image of the SiO_2_ nanoparticles used in this study. (**d**) An SEM image of uncoated PPTA fiber. (**e**) An SEM image of a PPTA fiber coated with UHMWPE and SiO_2_ nanoparticles. (**f**) EDS mapping of (**e**) showing the distribution of element Si on the coated fiber.

A high-temperature dip coating approach was used to coat the PPTA fibers with UHMWPE and SiO_2_ nanoparticles (Fig. 1a,b). First, SiO_2_ nanoparticles of ~ 200 nm (Fig. 1c) was synthesized by a Stöber method in ethanol/water solution^32^. The particle size distribution of the as-prepared SiO_2_ nanoparticle was determined using Nanomeasure software (inset of Fig. 1c). The synthesized SiO_2_ nanoparticles (30 wt.%) were homogeneously mixed with UHMWPE granules (Goodfellow Corp., Coraopolis, PA, USA). The mixture was then melted at ~200 °C and continuously stirred under argon atmosphere. To perform coating, PPTA single fibers (AR305722/1, DuPont Kevlar^_^29 from Goodfellow Corp., Coraopolis, PA, USA) were dipped into the mixture and slowly drawn from the melt. The coated fibers were cooled on a 60 °C hot plate for 10 min and stored at room temperature for further characterization and testing.

The morphology of the uncoated and coated fibers was examined by scanning electron microscopy (SEM). As shown in Fig. 1d, the uncoated fibers have smooth surface. The diameter of a single fiber is ~ 12 μm. After coating with UHMWPE and SiO_2_ nanoparticles, the diameter of the fiber increased to ~15 μm (Fig. 1e). The nanoparticles were homogeneously distributed, which is confirmed by the EDS mapping of Si (Fig. 1f).

**Figure 2:**
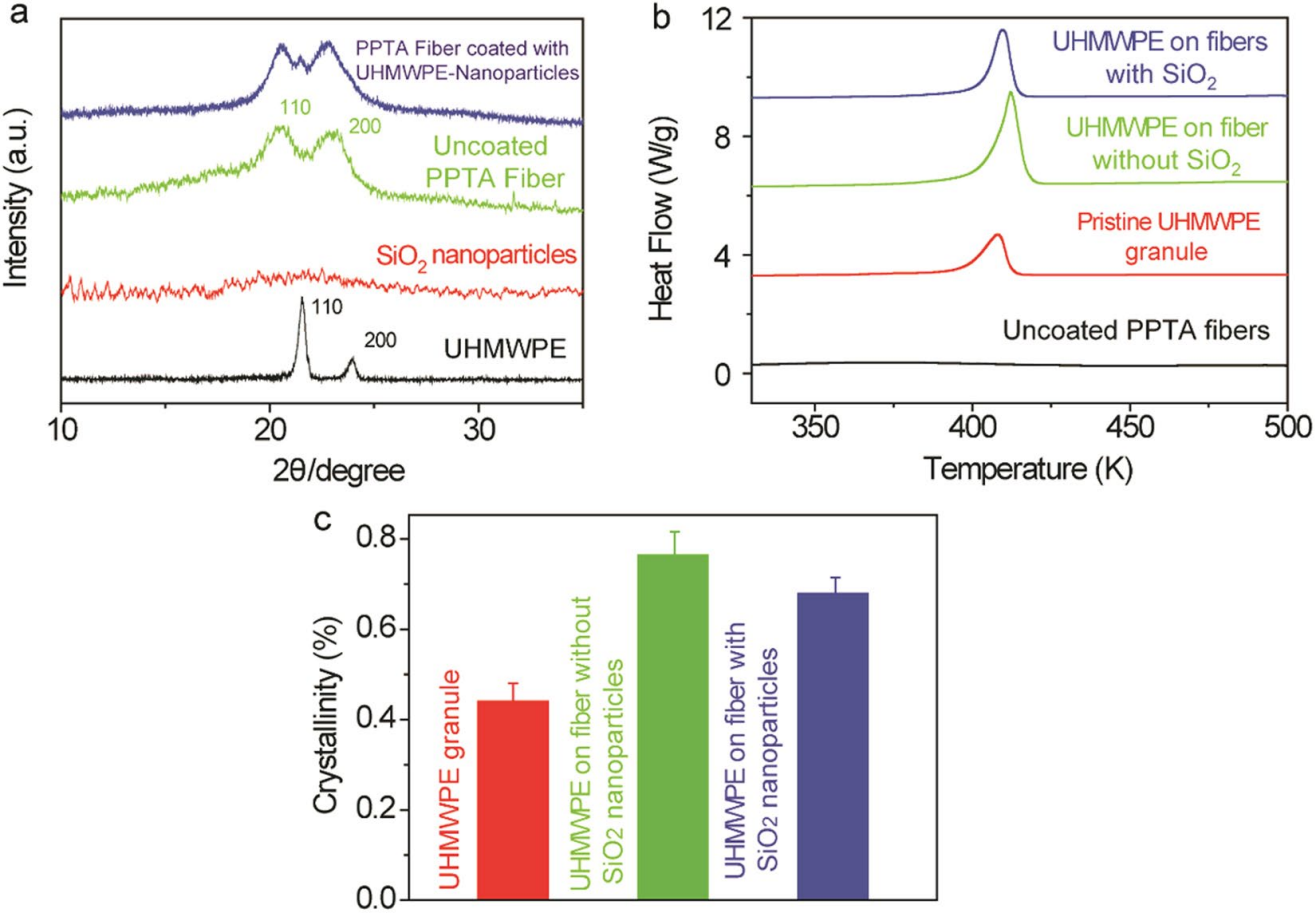
Structure of the uncoated, UHMWPE coated and UHMWPE-SiO_2_ coated PPTA fiber. (**a**) X-ray diffraction (XRD) profiles of uncoated, UHMWPE coated and UHMWPE-SiO_2_ coated PPTA fiber. (**b**) Differential scanning calorimetry (DSC) analysis of uncoated PPTA fiber, pristine UHMWPE granule, UHMWPE coated on PPTA fiber without nanoparticles and UHMWPE coated on PPTA fiber with nanoparticles. (**c**) The crystallinity of the pristine and coated UHMWPE was calculated by comparing the enthalpy of fusion between the theoretical value and the experimental data.

Figure 2a shows the x-ray diffraction profiles of the coated PPTA fiber and its constituents. For the pure UHMWPE granule material, two distinct peaks are observed at 21.44° and 23.99°, which correspond to the (110) and (200) planes, respectively^33^. No peak is observed from SiO_2_ nanoparticles, indicating their amorphous nature^34^. For uncoated PPTA fiber, the (110) and (200) peaks are observed at 20.49° and 23.11°, respectively^35^. Three distinct peaks are detected for the coated PPTA fibers: the (110) and (200) peaks of PPTA and the (110) peak of UHMWPE, which are respectively attributed to the fiber phase and the coated layer.

Differential scanning calorimetry (DSC) measurements were conducted to investigate the materials’ thermal behavior (Fig. 2b). Due to the mealting of UHMWPE, an endothermic peak around 411.9 K is observed for the UHMWPE-coated PPTA fiber. This melting point is 3.5 K higher than that of pure UHMWPE, which is 408.4 K. This increase of the melting point indicates the crystallinity of the UHMWPE is higher as coated on PPTA. It is likely that the surface of ordered PPTA polymer could serve as a template for the nucleation of UHMWPE and crystal grow^36^. On the other hand, by incorporationg SiO_2_ in the coating decreases the melting point of UHMWPE from 411.9 K to 409.4 K. This could imply that the existence of the nanoparticles could reduce the crystallinity of the UHMWPE phase. The addition of SiO_2_ nanoparticles can result in formation of small imperfect crystallites, which in turn can hinder the mobility of UHMWPE chains during the crystallization process^37^. Due to the good thermal stability of PPTA, no peaks are observed between room temperature and 500 K for the uncoated PPTA fiber.

The crystallinity of the UHMWPE phase can be estimated from the ratio between the measured and theoretical values of the enthalpy of fusion^38^. The degree of crystallinity of the UHMWPE coated on PPTA fiber was estimated to be 79%, which decreased to approximately 67% after incorporation of SiO_2_ nanoparticles (Fig. 2c). In contrast, the degree of crystallinity of the raw UHMWPE granules was about 42%. This result is consistent with the observed changes in the melting point. The increased crystallinity of the coating suggests that the PPTA surface could promote the ordering of the UHMWPE coating, which induced a higher crystallization.

**Figure 3:**
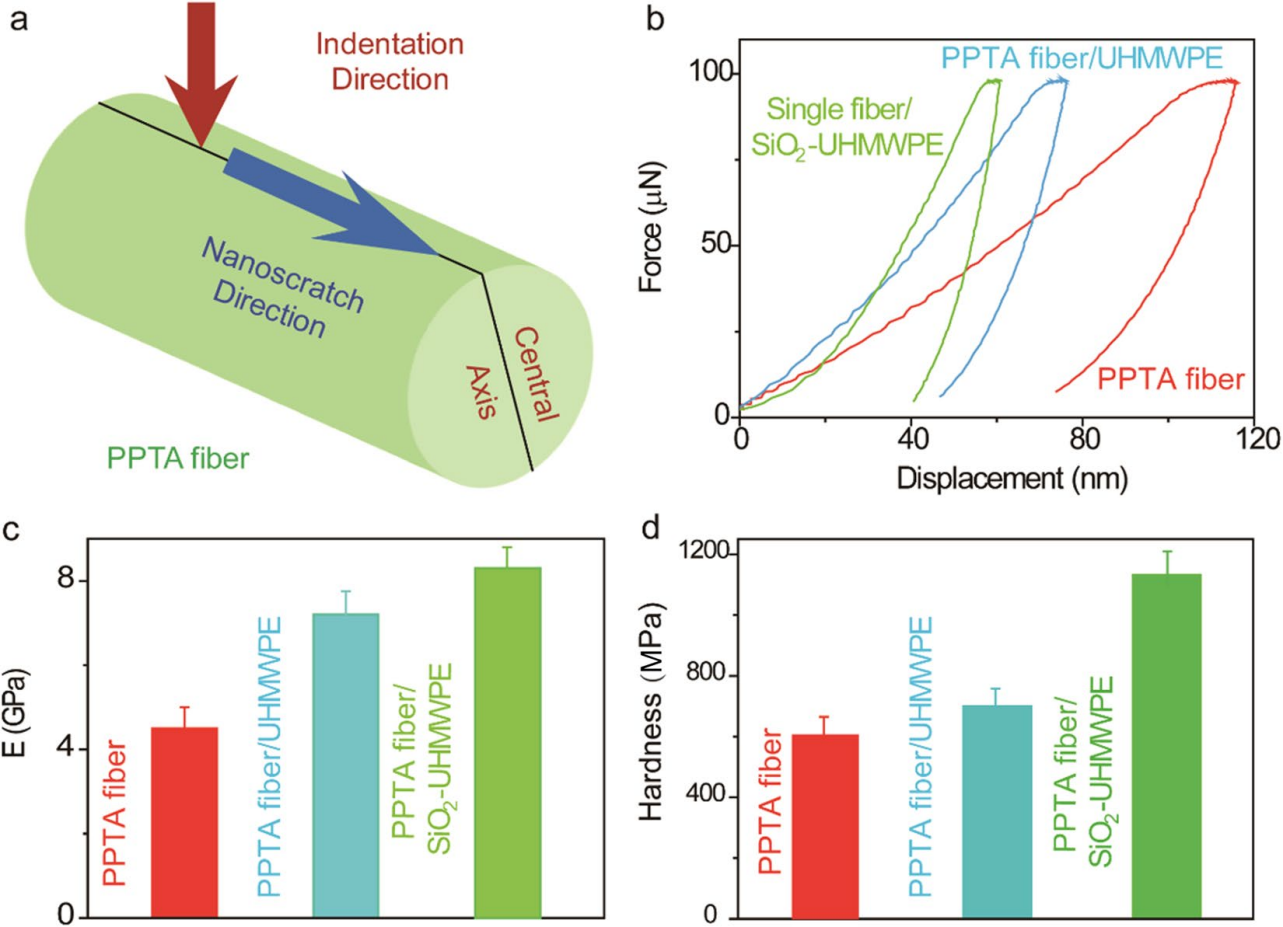
Mechanical properties of PPTA fibers with and without coatings at the surface region. (**a**) Schematic illustration of nanoindentation and scratch testing on single fibers. (**b**) Representative force-displacement curves during nanoindentation testing. (**c**) Young’s modulus of uncoated, UHMWPE coated and UHMWPE-SiO_2_ coated PPTA fibers. (**d**) Hardness of uncoated, UHMWPE coated and UHMWPE-SiO_2_ coated PPTA fibers.

The mechanical properties along the transverse direction were investigated by two characterization method. The first focused more at the surface region by conducting nanoindentation on single fibers (Fig. 3a). The measurements were conducted in the force-control mode in which the maximum force applied on the fibers is 100 µN (using a Berkovich tip with the diameter about 1.5 µm)^39^. Fig. 3a shows some typical loading-unloading curves for uncoated PPTA fibers, PPTA fibers coated with UHMWPE, and PPTA fibers coated with UHMWPE and SiO_2_ nanoparticles. The Young’s modulus was calculated from the unloading curves: ~ 4.5 GPa for uncoated PPTA fibers, ~7.2 GPa for UHMWPE coated PPTA fibers, and ~8.3 GPa for UHMWPE/SiO_2_ nanoparticle coated PPTA fibers. These results indicate that the UHMWPE coating on the PPTA fibers can effectively enhance its Young’s modulus in the transverse direction, which is likely due to high stiffness of the highly crystalline UHMWPE coating. Further enhancement of Young’s modulus by embedding SiO_2_ nanoparticles in UHMWPE (30 wt.%) is due to the high Young’s modulus of the ceramic phase (~70 GPa). In addition to modulus, the hardness also increased from ~600 MPa to ~1100 MPa after coating with UHMWPE/SiO_2_ nanoparticles. The simultaneous increase in fiber stiffness and the hardness could increase the fiber/fabric’s bending resistance and their ability to absorb more impact energy.

**Figure 4:**
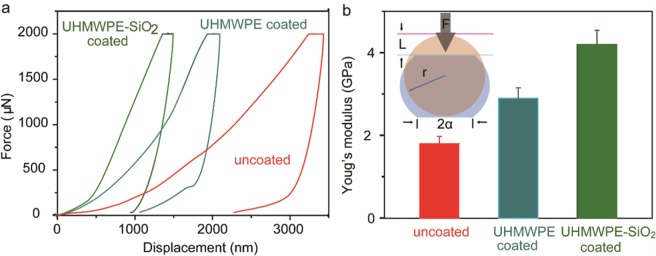
Mechanical properties of whole PPTA fibers with and without coatings. (**a**) Force-displacement curves of uncoated, UHMWPE coated and UHMWPE-SiO_2_ coated PPTA fiber. (**b**) The compressive Young’s modules of the whole uncoated, UHMWPE coated and UHMWPE-SiO_2_ coated fibers. Inset: Scheme illustration of a transverse compressively loaded fiber.

In addition, to further clarify the effect of the coating on the mechanical properties along the transverse direction of the whole fiber, we adopted a large flat punch tip with the diameter of 100 μm to do the indentation. The scheme of a transverse compressively loaded fiber is shown in the inset of Fig. 4b. The flat punch tip was forced downward on the top of the fiber. We recorded the compressive force and vertical displacement during the indentation. Figure 4a shows the force-displacement curves of uncoated, UHMWPE coated and UHMWPE-SiO_2_ coated PPTA fibers. According to the literatures^40,41^ the compressive behavior of the fiber can be approximately considered linear elastic in its transverse direction at an infinitesimal deformation range. A relation was derived between compressive load and deflection based on a classical stress-field solution^42,43^ for this plane strain problem and the transversely isotropic constitutive relations in terms of nominal stress and nominal strain:

$$\partial =\frac{{\rm{4}}\sigma }{\pi {\alpha }^{2}}[(-\frac{{\upsilon }_{12}}{{E}_{1}}-\frac{{{\upsilon }^{2}}_{31}}{{E}_{3}})(\sqrt{{\alpha }^{{\rm{2}}}+{{\rm{r}}}^{{\rm{2}}}}-r)r+(\frac{1}{{E}_{1}}-\frac{{{\upsilon }^{2}}_{31}}{{E}_{3}}){\alpha }^{{\rm{2}}}(\frac{\sqrt{{\alpha }^{{\rm{2}}}+{{\rm{r}}}^{{\rm{2}}}}+r}{\alpha })]$$

$${\rm{with}}\,\alpha =\sqrt{\frac{{\rm{8}}\sigma {{\rm{r}}}^{{\rm{2}}}}{\pi }(\frac{1}{{E}_{1}}-\frac{{{\upsilon }^{2}}_{31}}{{E}_{3}})},\,\,\sigma =\frac{F}{2r}\,\,\,{\rm{and}}\,\,\,\partial =\frac{L}{2r}$$

where *E*_1_ and *E*_3_ are transverse and longitudinal Young’s moduli, respectively, which are constants within small deformation. *υ*_12_ and *υ*_31_ are transverse and longitudinal Poisson’s ratios, respectively. α is half of the width of the contact zone. r is the radius of the fiber specimen. σ is nominal transverse stress, ∂ is nominal transverse strain. F stands for the transverse compressive load per unit length along the fiber axial direction, and L the transverse displacement. With the parameters provided above, the compressive Young’s modules of the uncoated, UHMWPE coated and UHMWPE-SiO_2_ coated fibers was estimated to be ~1.8 GPa, ~2.9 GPa and ~4.2 GPa, which is depicted in Fig. 4b. The enhancement of the Young’s modulus of the whole fiber along transverse direction further confirms the beneficial effect by UHMWPE and SiO_2_ coating, which made it very promising as a new generation of high-performance polymer engineering materials.

**Figure 5:**
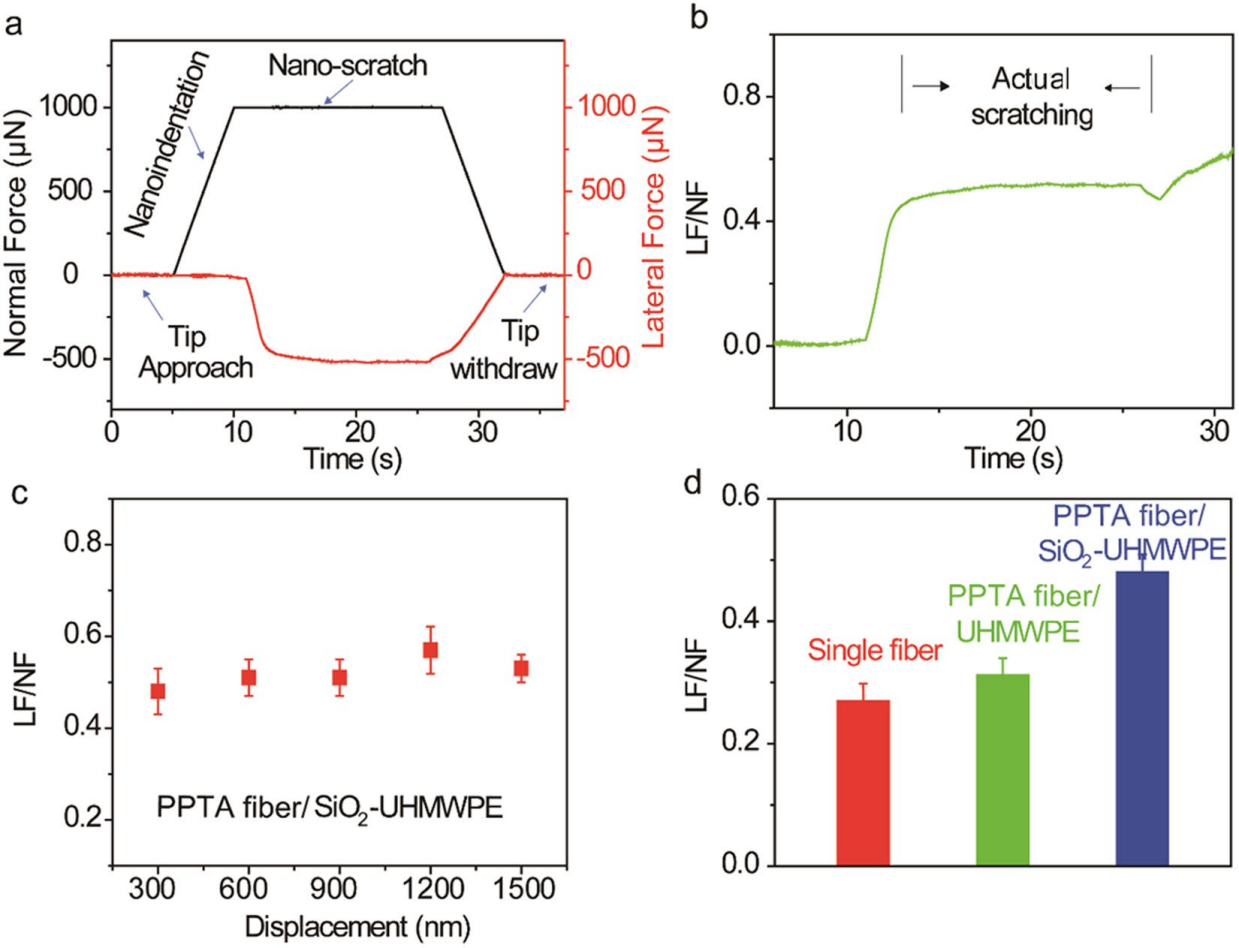
Surface friction characterization. (**a**) Typical force-time curve during nanoscratch testing. (**b**) Variation of the ratio between the lateral force and the normal force (friction coefficient) as a function of testing time. (**c**) Friction coefficient of UHMWPE-SiO_2_ coated PPTA fiber as a function of initial indentation depth ranging from 300 nm to 1500 nm. (**d**) Friction coefficients of uncoated, UHMWPE coated and UHMWPE-SiO_2_ coated PPTA fibers.

Next, to evaluate the surface frictional behavior of the fibers, the nano-scratch capability of the nanoindentation (Fig. 3a) has been used. Figure 5a reveals the normal and lateral forces on a UHMWPE-SiO_2_ coated PPTA fiber during the nanoscratch test. By calculating the ratio between the lateral force and the normal force (LF/NF) from Fig. 5a, the friction coefficient curve of the sample was obtained (Fig. 5b)^44–51^, which shows a value of ~ 0.52 in the actual scratching period (10–25 sec). Figure 4c shows the effect of indentation depth on the friction coefficient, which indicates little variation between 300 nm and 1500 nm. Figure 5d compares the friction coefficient of coated and uncoated fibers. The uncoated fiber exhibit a friction of coefficient of ~0.27, which is comparable to a previous study where the yarn-yarn friction coefficient of PPTA fibers were found to be between 0.2–0.3^47^. While coating PPTA fibers with UHMWPE only provides marginal improvement, incorporating SiO_2_ nanoparticles induces significant enhancement of the friction coefficient, which may be related to two contributing factors. On one hand, nanoparticles can increase surface roughness, which in turn can increase the adhesion friction coefficient. On the other hand, the gouging effect of the nanoparticles during the sliding of fibers can raise significantly the ploughing friction coefficient. This result implies that UHMWPE/SiO_2_ nanoparticle coating has good potential to improve the surface friction of PPTA fabrics for the body armor applications.

**Figure 6:**
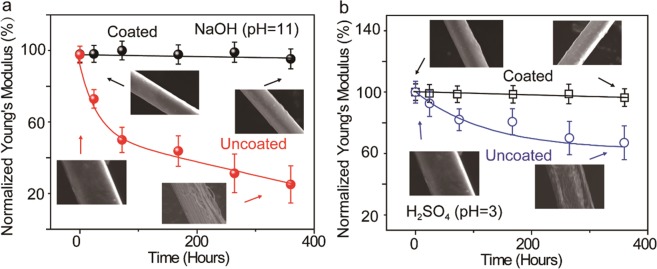
Effect of harsh environment on the mechanical behavior of fibers with and without coating. (**a**) Relative values of Young’s modulus as a function of immersion time in NaOH solution (pH = 11). Inset: SEM images of the uncoated and coated fibers before (left) and after 350 hrs of treatment (right). (**b**) Relative values of Young’s modulus as a function of immersion time in H_2_SO_4_ (pH = 3). Inset: SEM images of the pristine PPTA and coated fiber before and after 350 hrs of treatment.

In addition to enhancing the mechanical properties in the transverse direction, improving the robustness of PPTA fibers against chemical attack is also highly desired. In many protective applications, PPTA fibers are exposed to harsh environments, including acidic and alkaline conditions which, over time, can degrade their mechanical properties. Although the inter-molecular hydrogen bonds formed between the carbonyl groups and -NH centers as well as the aromatic stacking interaction between adjacent strands provide superior longitudinal mechanical properties and some chemical durability to PPTA fibers, these bonds may degrade under certain acidic/alkaline conditions (e.g. H_2_SO_4_, NaOH), resulting in reduced mechanical performance. In order to evaluate the protective effect of the UHMWPE/SiO_2_ nanoparticle coating, coated PPTA fibers along with uncoated fibers were immersed in either H_2_SO_4_ or NaOH, and the Young’s modulus of these fibers was monitored as a function of immersion time (Fig. 6).

Uncoated PPTA fibers exhibit continuous decrease of Young’s modulus in the NaOH solution (pH = 11). The initial Young’s modulus of the uncoated fiber was approximately 5 GPa, and decreased to more than 70% after 350 hrs of alkaline treatment. Similar trend was observed for acid treatment. Figure 6b shows that Young’s modulus of uncoated PPTA fibers decreased by about 40% after 350 hrs of immersion in H_2_SO_4_ (pH = 3). This decrease is likely the result of fiber degradation due to breakage of hydrogen bonds of PPTA fibers, as indicated by subsequent SEM analysis (Inset of Fig. 6a,b). This result implies that the performance of uncoated PPTA fibers or fabrics would degrade over time under such harsh environments. In contrast, with UHMWPE coating, the Young’s modulus of PPTA fibers remains relatively unchanged up to 350 hrs (the longest immersion time included in this study). The morphologies of the coated fiber kept almost unchanged after NaOH or H_2_SO_4_ treatments while the pristine fibers were corroded and damaged. This result demonstrates the UHMWPE coating can not only improve the Young’s modulus of PPTA fibers but also enhances its resistance to chemical attack.

In summary, this paper presents a facile polymer dip-coating strategy to cover the surface of PPTA fibers with a layer of ultra-high molecular weight polyethylene (UHMWPE). As a result, the transverse mechanical properties of the fiber are significantly improved. Furturemore, the effect of incorporating SiO_2_ nanoparticles into the UHMWPE phase has been explored, showing a remarkable increase of friction coefficient (nearly by 100%.) The Young’s modulus and the hardness of the PPTA fibers with the UHMWPE/SiO_2_ coating also exhibit significant enhancement. Moreover, this coating can shield the PPTA fibers from some chemical attacks and alleviate the loss in their mechanical performance. These results demonstrate the UHMWPE/SiO_2_ coating could simultaneously enhance multiple mechanical properties of PPTA fibers and extend their applicability in protective applications.

## Methods

### Fabrication of SiO_2_-incorporated UHMWPE coating on PPTA fibers

SiO_2_ nanoparticles with ~200 nm were synthesized using Stöber method in ethanol solution. The average particle size of each SiO_2_ nanoparticle batch was assessed using the Nanomeasure software. The resulting SiO_2_ nanoparticles (30% in weight percentage) were mixed mechanically with UHMWPE granules (Goodfellow Corp., Coraopolis, PA, USA). The mixture was melted in a mold and a clear viscous solution was obtained with homogeneous stirring in an argon atmosphere at ~200 °C. The commercially available PPTA fibers was dipped into the mixture and then been slowly drawing out from the solution for the coating. The coated fibers were then cooled on a 60 °C hot plate for 10 min for further use.

### Morphology and structure characterization

SEM images were taken using a FEI Quanta450FEG scanning electron microscope. X-ray diffraction analysis was carried out using a Rigaku CCD diffractometer with Cu-Kα radiation (λ = 1.54184 Å). Differential Scanning Calorimeter (DSC) scans were run between 300 K and 500 K using a TA Instruments DSC2500 thermal system purged with nitrogen. The sample weight was around 10 mg.

### Mechanical properties characterization

A Hysteron TI 980 TriboIndenter was used to measure displacement-force curves during indentation experiments. Measurements were performed in displacement control mode, with a maximum indent depth of approximately 100 nm. A drift correction was performed prior to indentation. The loading rate was 20 nm s^−1^. Holding time was 5 seconds. The Young’s modulus was obtained from the force-displacement behavior as follows:

$$\frac{1}{{E}_{r}}=\frac{(1-{v}^{2})}{E}+\frac{(1-{v}_{i}^{2})}{{E}_{i}},\,\,{E}_{r}=\frac{\sqrt{\pi }}{2\beta }\frac{S}{\sqrt{A}}$$

where *E*_*r*_ is the reduced modulus, *E*_*i*_ is the Young’s modulus of the indenter, ν and ν_i_ are the Poisson’s ratio of the specimen and the indenter, respectively^39^. *A* is the contact surface area at peak load, *S* is the initial unloading contact stiffness calculated with $S=\frac{dP}{dh}$, and *β* is an indenter geometry dependent dimensionless parameter. Typically, 5 indentations were made at each depth on each fiber type.

Nano-scratch is performed by applying a normal load in a controlled fashion on the Z-axis of the transducer while measuring the force required to move the probe laterally across the sample. The friction coefficient is defined as the ratio between the lateral force and the normal force (LF/NF). In the first five seconds, the probe was moved backward from the center point and slowly approaching to the sample surface without normal force applied. After that, a nanoindetation was applied on the sample surface with a specific normal force from five seconds to ten seconds without lateral force. The tip was then moved forward along the central axis for the nanoscratch test by sustaining the same normal force from ~10 seconds to ~25 seconds (actual scratching period). As a result, the lateral force was recorded during the scratch. Finally, the lateral force and the normal force were withdrawn gradually by the transducer to finish the nanoscratch. By calculating the ratio between the lateral force and the normal force, the friction coefficient curve of the sample was obtained. Typically, 5 scratches were made at each depth on each fiber type.

### H_2_SO_4_ and NaOH treatments

The PPTA fibers were aligned on a triangle frame to prevent shrinkage and distortion. The frame was then immersed into H_2_SO_4_ solution (pH = 3) or NaOH solution (pH = 1 1) for a certain amount of time. After that, the resulting PPTA fibers were dried in an oven at 80 °C for 30 min for further characterization of the morphology and and testing of mechanical properties.
